# Tailoring the Health-Promoting Potential of Protein Hydrolysate Derived from Fish Wastes and Flavonoids from Yellow Onion Skins: From Binding Mechanisms to Microencapsulated Functional Ingredients

**DOI:** 10.3390/biom10101416

**Published:** 2020-10-07

**Authors:** Leontina Grigore-Gurgu, Oana Crăciunescu, Iuliana Aprodu, Carmen Alina Bolea, Andreea Iosăgeanu, Brîndușa Alina Petre, Gabriela Elena Bahrim, Anca Oancea, Nicoleta Stănciuc

**Affiliations:** 1 Faculty of Food Science and Engineering, Dunarea de Jos University of Galati, 111 Domnească Street, 800201 Galați, Romania; Leontina.Gurgu@ugal.ro (L.G.-G.); Iuliana.Aprodu@ugal.ro (I.A.); Carmen.Bolea@ugal.ro (C.A.B.); Gabriela.Bahrim@ugal.ro (G.E.B.); 2 National Institute of Research and & Development for Biological Sciences, 296 Splaiul Independentei, 060031 Bucharest, Romania; oana_craciunescu2009@yahoo.com (O.C.); andreea.iosageanu@gmail.com (A.I.); oancea.anca@gmail.com (A.O.); 3 Faculty of Chemistry, Alexandru Ioan Cuza University of Iasi, 700506 Iaşi, Romania; brindusa.petre@uaic.ro; 4 Center for Fundamental Research and Experimental Development in Translation Medicine–TRANSCEND, Regional Institute of Oncology, 700483 Iaşi, Romania

**Keywords:** bioactives, peptides, fish waste, flavonoids, yellow onion skins, biological properties, hydrolysis, microencapsulation

## Abstract

This study focuses on combining different bioprocessing tools in order to develop an in-depth engineering approach for enhancing the biological properties of two valuable food by-products, namely fish waste and yellow onion skins, in a single new bioactive formulation. Bone tissue from phytophagous carp (*Hypophthalmichthys molitrix*) was used to obtain bioactive peptides through papain-assisted hydrolysis. The peptides with molecular weight lower than 3 kDa were characterized through MALDI-ToF/ToF mass spectrometry and bioinformatics tools. As a prerequisite for microencapsulation, the ability of these peptides to bind the flavonoids extracted from yellow onion skins was further tested through fluorescence quenching measurements. The results obtained demonstrate a considerable binding potency with a binding value of 10^6^ and also the presence of one single or one class of binding site during the interaction process of flavonoids with peptides, in which the main forces involved are hydrogen bonds and van der Waals interactions. In the freeze-drying microencapsulation process, an efficiency for total flavonoids of 88.68 ± 2.37% was obtained, considering the total flavonoids and total polyphenols from the powder of 75.72 ± 2.58 quercetin equivalents/g dry weight (DW) and 97.32 ± 2.80 gallic acid equivalents/g DW, respectively. The 3-(4,5-dimethylthiazol-2-yl)-2,5-diphenyltetrazolium bromide (MTT) test on the L929 cell line cultivated in the presence of different concentrations of microencapsulated samples (0.05–1.5 mg/mL) proved no sign of cytotoxicity, the cell viability being over 80% for all the samples.

## 1. Introduction

The fish trade accounts for approximately 140 million tons of fish production, including aquaculture, out of which 110 million is for human consumption. The head, skin, trimmings, fins, frames, viscera, and roe that result during fish processing are underutilized protein rich by-product materials that are discarded without recovery attempts, representing more than 60% of biomass [1]. Nowadays, the interest in producing fish hydrolysates has increased due to the value-added potential of the resulting peptides that can be used as functional ingredients for commercial food products [2]. Different reports have shown excellent physicochemical and functional properties for fish protein hydrolysates, such as antioxidant activity against free radicals [3,4], antihypertensive pharmacological agents, specifically as inhibitors of the angiotensin-I converting enzyme (ACE) [5], anti-proliferative and anti-inflammatory, antimicrobial, cytomodulatory and immuno-modulatory functions [6].

On the other hand, the food industry produces a large amount of onion wastes, which are valuable due to the high amount of bioactive compounds, especially flavonoids. The reuse of onion by-products in high-value functional and healthy ingredients may offer the opportunity to valorize these low-cost food by-products in economically interesting perspectives, exploiting their potential applications in functional foods, supplements, cosmetics, and nutraceutical products. This comes in a context in which consumers increasingly refuse synthetic food supplements, expressing their preferences for ingredients from natural sources with beneficial effects on health [7,8]. It has been reported that the major by-products resulting from the industrial peeling of onion bulbs are dried skin, the outer two fleshy leaves, and the top and bottom bulbs, which are not edible and are removed before processing [9]. Onion wastes, due to a rapid phytopathogens growth and to their strong unique smell, are not suitable for fodder or landfill disposal [7]. Therefore, the development of strategies to valorize these by-products is immediately required, with benefits for both processors and consumers, while significantly reducing the negative impact on the environment. The main phytochemicals found in onions are phenolic acids, anthocyanins, cepaenes, thiosulfinates, and flavonoids [10], whereas the major bioactive compounds found in skins are phenolics as quercetin and quercetin glycosides [11]. Quercetin is of great interest due to its antimicrobial and antioxidant activity, which are strongly related with a role in cancer prevention, inflammatory disorders, cardiovascular diseases [12], anti-inflammatory activity, antihistamine effect, allergy medication, anticancer and antivirus activities, and reducing blood pressure in hypertensive subjects [7].

The objective of this study was to employ an integrative approach for a multi-directional reuse of two different food by-products, namely fish waste and yellow onion skins, which might open new perspectives for developing diet supplements, with health-promoting activity. Two analytical approaches were performed, such as the hydrolysis of fish waste to obtain bioactive peptides and the ultrasound-assisted extraction of phytomolecules from yellow onion skins. The hydrolysates were characterized in terms of peptides profile, using chromatographic methods and mass spectrometry, whereas onion skins extract was analyzed in terms of global flavonoids, polyphenolic contents, and antioxidant activity. A fundamental investigation relying on the use of fluorescence quenching was carried out to detail the binding parameters associated to the interaction between flavonoids and fish peptides as a prerequisite for microencapsulation. The flavonoids from the extract were microencapsulated in fish waste peptides with low molecular weight, using inulin and chitosan as adjuvants by freeze-drying. The powder was tested for microencapsulation efficiency, phytochemicals, antioxidant activity, cytotoxicity, and cells viability. Our results give grounds for further studies on the interplay between the biological activity of multiple polyphenolic compounds and peptides, thus highlighting its contribution to the antioxidative and cytostatic activities as functional ingredients.

## 2. Materials and Methods 

### 2.1. Chemicals

Quercetin, 2,2-diphenyl-1-picrylhydrazyl (DPPH), 6-hydroxy-2,5,7,8-tetramethylchromane-2-carboxylic acid (Trolox), ethanol, sodium hydroxide, Folin–Ciocalteu reagent, gallic acid, gelatin type A, 3-(4,5-dimethylthiazol-2-yl)-2,5-diphenyltetrazolium bromide (MTT), inulin, and chitosan were obtained from Sigma Aldrich Steinheim, Germany. For cell culture experiments, Minimum Essential Medium (MEM), fetal bovine serum (FBS), L-glutamine, mixture of antibiotics (penicillin, streptomycin, neomycin: PSN) and Neutral red (NR) were purchased from Merck (Germany). The NCTC clone L929 cell line of mouse fibroblasts was from ECACC (Sigma-Aldrich, Germany).

### 2.2. Extraction of Flavonoids from Yellow Onion Skins

Yellow onions were purchased from a local market (Galati, Romania) in June 2018. The outer layers of onions were collected, cleaned with distilled water, dried, milled, and used for further analysis. The flavonoid extract was obtained by mixing 50 g of the sample with 450 mL of 70% ethanol solution and glacial acetic acid (ratio 9:1, *v*/*v*). The extraction was performed at 40 °C for 30 min, using a sonication water bath, followed by 10 min centrifugation at 5000× *g* and 4 °C. The supernatant was collected, and the same extraction procedure was repeated three times. After extraction, the collected supernatants were pooled together and concentrated under reduced pressure at 40 °C. The obtained extract was dissolved in ultrapure water, characterized, and used for microencapsulation experiments.

### 2.3. Fish Waste Material Preliminary Preparation

To obtain bioactive peptides, bone tissue from phytophagous carp (*Hypophthalmichthys molitrix*) was used. The preparation of the raw material consisted of crushing the bone tissue residues (approximatively 200 g) and then homogenizing them in distilled water (1:1) using a mixer to obtain a paste. In order to remove calcium, the sample was preliminary treated with 200 mL of 1% EGTA in 0.05 M Tris buffer (pH 7.0) with stirring at room temperature for 8 h. The next day, a delipidation of the resulting paste was performed in 200 mL of 0.05 M Tris buffer, pH 7.0 by incubation at 55 °C, for 4 h and at 90 °C, for 15 min. After cooling, the mixture was kept in the refrigerator overnight to separate the residual fats. Centrifugation was performed for 20 min at 10.000× *g* and at 4°C, whereas the precipitates were combined.

### 2.4. Enzymatic Hydrolysis of Fish Waste 

Homogeneous white paste (150 g) was subjected to enzymatic hydrolysis at 55 °C, pH 5.5, by incubating for 6 h the dry tissue with 300 mL of distilled water containing papain in a water bath with stirring at 350 rpm for to improve the hydrolysis reaction. The enzyme/dry tissues ratio was 1:25 (g/g). For enzyme inactivation, the solution was incubated at 100 °C for 5 min. After cooling, the solution was filtered at the vacuum pump and the supernatant containing bioactive peptides was fractionated by centrifugal ultrafiltration through YM3 membranes with a 3 kDa molecular weight cut off (Amicon), at 7500× *g* for 25 min. The resulting low molecular weight peptides fraction was stored at −20 °C until further use in binding and microencapsulation experiments.

### 2.5. Bioactive Peptides Identification and Characterization

The protein content was determined using bicinchoninic acid (BCA) assay, and a standard curve was created with 0–3 mg/mL gelatin type A as standard protein. The peptides mass was analyzed by MALDI-ToF /ToF mass spectrometry using MALDI-ToF/ToF UtrafleXtreme equipment (Bruker Daltronik, Bremen, Germany). The main low molecular weight peptide fractions obtained after enzymatic hydrolysis were separated, and their molecular masses were identified. For MALDI ToF/ToF measurements, similar conditions, matrices, spectra parameters and external peptide mix calibrators were employed, as previously reported by Condurache et al. [13]. The amino acids sequence of the peptides resulting from collagen hydrolysis was further predicted using the information available in the Uniprot database. The UniProtKB entries A0A077B3P8 (gene COL1A1) and A0A2H4ZEX8 (gene COLlA2) encoding the collagen type I α1 and collagen type I α2 from *Hypophthalmichthys molitrix* (Silver carp) were considered. The potential cleavage sites of papain were identified considering that the enzyme is able to recognize and preferentially hydrolyzes the peptide bonds located in the vicinity of arginine or lysine residues preceded by one of the following hydrophobic amino acids: alanine, valine, leucine, isoleucine, phenylalanine, tryptophan, or tyrosine. The papain will not cleave the peptide bonds if the arginine or lysine residues are followed by valine residues.

### 2.6. Heat Treatment 

The low molecular weight peptides (0.15 mL) were filled in Eppendorf tubes (Eppendorf AG, Hamburg, Germany). The thermal treatment experiments were conducted in a thermostatic water bath (Digibath-2 BAD 4, Raypa Trade, Barcelona, Spain) at temperatures ranging from 25 to 95 °C for 15 min. As previously tested by our group, it has been considered that the holding time chosen to maintain the solutions at constant temperature is long enough to ensure structural rearrangements within the peptide chains. The tubes were immediately cooled in iced water to prevent further thermal denaturation. 

### 2.7. Quenching of Bioactive Peptides with Yellow Onion Skins Extract

The (un)-heat-treated samples (0.100 mL) were diluted in 3 mL of in 50 mM Tris-HCl buffer solution at pH 7.7 and titrated by the successive addition of the yellow onion skin extract (2 mg of extract prepared in 1 mL of distilled water). The excitation wavelength was set at 295 nm, while the width of both excitation and emission slits were set to 10 nm. The Stern–Volmer constants, binding constants, and number of binding sites were calculated as previously reported [14]. All fluorescence spectra were recorded on a LS-55 luminescence spectrometer (Perkin Elmer Life Sciences, Shelton, CT, USA) equipped with the software Perkin Elmer FL Winlab. The inner filter effect was corrected as described previously by Horincar et al. [15].

### 2.8. Microencapsulation of the Flavonoidic Extract in Bioactive Fish Peptides

The concentrated extract (3 g) obtained from yellow onion peels was dissolved in 200 mL of low molecular weight fish peptides solution, stirred for 2 h at 650 rpm and 40 °C, and then centrifuged at 9000× *g* for 20 min at 4 °C in order to collect the supernatant. In the resulting supernatant, 2 g of inulin and 4 g of chitosan were dissolved by stirring at 40 °C, followed by coacervation up to pH 4.5. Then, the mixture was subjected for 48 h to the freeze-drying process at −42 °C (CHRIST Alpha 1-4 LD plus, Osterode am Harz, Germany), under a pressure of 10 Pa. Afterwards, the powder was collected, packed in metallized bags, and kept at 4 °C for further analysis.

### 2.9. Characterization of the Extract and of Microencapsulated Powders

The yellow onion skin extract was characterized in terms of flavonoids, polyphenolic contents, and antioxidant activity by using the aluminum chloride, Folin–Ciocâlteu, and DPPH methods, respectively, as described by Milea et al. [16]. In order to characterize the powders, the protocols described by Oancea et al. [17] were employed for assaying the encapsulation efficiency, total flavonoids, total polyphenol contents, and antioxidant activity.

### 2.10. Cell Culture and T

Cell culture tests were performed according to SR EN ISO 10993-5 [18] for medical device cytotoxicity using the direct contact method. Briefly, L929 mouse fibroblasts were cultured as adherent cells in T25 flasks, in MEM supplemented with 10% FBS and 1% PSN, in humid atmosphere with 5% CO_2_ at 37 °C. The passages were performed every 4 days. A stock solution of 1.5 mg/mL microencapsulated material was prepared in MEM supplemented with 10% FBS and 1% PSN and sterile filtered through membranes with 0.22 µm pore size. For cytotoxicity experiments, the cells were seeded in 24-well culture plates at a density of 4 × 10^4^ cells/mL and incubated in 5% CO_2_ atmosphere at 37 °C for 24 h in order to adhere to the polystyrene surfaces. Then, the culture medium was replaced by serially diluted stock solution to get sample concentrations in the range of 0.05–1.5 mg/mL, and the plates were incubated in standard conditions for 24 h. Cells cultivated in MEM without a sample represented the negative control, while cells treated with 50 µM H_2_O_2_ served as positive control. The experiment was conducted in triplicate.

### 2.11. In Vitro Cytotoxicity Tests

The quantitative evaluation of samples cytotoxicity was performed by MTT assay, as previously described by Crăciunescu et al. [19]. After the incubation period, the culture medium was removed, 0.25 mg/mL of MTT solution in MEM was added in each well, and plates were incubated at 37 °C for 3 h. Then, the resulted formazan crystals were solubilized in isopropyl alcohol by gentle shaking and the optical density (OD) of the colored solution was registered at a wavelength of 570 nm, using a microplate reader (BMG Labtech, Germany). The measured OD value is directly proportional to the number of viable cells. Cell viability was calculated as a percentage from untreated cells (control), using Equation (1):

Cell viability (%) = OD_sample_/OD_control_ × 100 (1)

The qualitative evaluation of samples cytotoxicity was performed by a light microscopy examination of cell morphology after 24 h of cultivation in the presence of the microencapsulated sample. The cells were washed, fixed in methanol, and stained with Giemsa. The cell morphology was observed in micrographs acquired at an Axio Observer microscope (Carl Zeiss, Germany) equipped with a digital camera driven by soft.

### 2.12. Statistical Analysis

All experimental measurements were performed at least in triplicate, and the results are presented as mean value ± standard deviation (SD). The one-way analysis of variance (ANOVA) and Tukey’s test with a 95% confidence interval was applied using Minitab 18 software to identify significant differences among fluorescence spectroscopy results. Statistical analysis of the cell culture results was performed using two-tailed, two-sample equal variance Student’s t-test. Differences were considered statistically significant at *p* ≤ 0.05 and *p* ≤ 0.01 as minimal levels of significance.

## 3. Results

### 3.1. Peptides Identification and Characterization

Low molecular weight peptides from fish waste were obtained by enzymatic treatment followed by centrifugal ultrafiltration, resulting a yield of 7.08%. Furthermore, the results were correlated with MALDI-TOF experiment within database searches using bioinformatic tools aiming to identify the peptides that correspond to the spectrum [20]. Therefore, in order to simulate the complete papain-assisted hydrolysis of the main proteins found in fish bones, the amino acids sequence of collagen type I α1 and collagen type I α2 was taken from UniProtKB database. The collagen type I α1 hydrolysis resulted in a total of 36 peptides with molecular weights ranging from 360.45 to 21392.42 Da, whereas the hydrolysis of collagen type I α2 generated a mixture of 37 peptides with molecular weights ranging from 561.59 to 8637.33 Da. In agreement with the experimental procedure, the peptides with a molecular weight lower than 3 kDa were further analyzed in detail.

**Figure 1:**
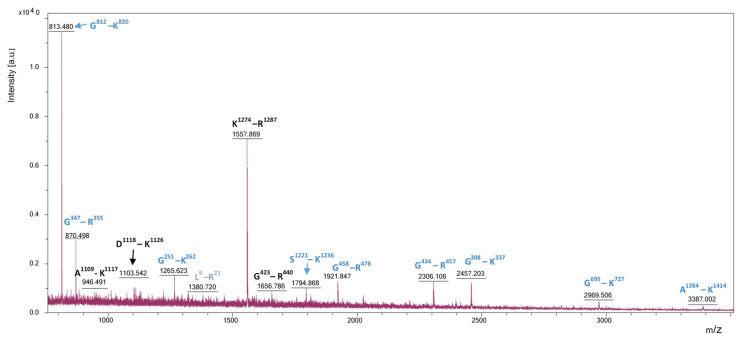
MALDI-TOF mass spectrum obtained after hydrolysis with papain and DTT reduction of the fish waste sample. Blue and black indicate the peptides that correspond with those resulted from the hydrolysis of collagen type I α1 and α2, respectively.

In addition, the MALDI-TOF spectrum of the hydrolyzed fish waste sample showed 14 peptides, which were identified with a total mass accuracy of <0.5 Da. This experiment highlighted that ten of the peptides resulted from collagen type I α1 hydrolysis (Figure 1, blue color) and the other four resulted from the collagen type I α2 (Figure 1, black color).

The properties of the peptides identified through bioinformatics tools matching the peaks from the MALDI-TOF mass spectrum (Figure 1) are presented in Table 1.

The molecular weight of the peptides, D1118–K1126 and G251–K262 were identified taking in account two different adducts [M + Na + K]^+^, while for G423–R440, G458–R478, G434–R457, and G695–K727, it was considered a doubly sodiated ion [M + 2Na]^+^ and a doubly potassiated ion [M + 2K]^+^ for S1221–K1236, respectively. Moreover, for two other peptides L9–R21 and A1384–K1414, an [−5Da] modification was observed due to a possible bridge formed in the sequence, between R and H amino acids, while for K1274–R1287, the MALDI-TOF spectrum exhibits a possible N-(beta-Aspartyl)-Lysine crosslinking (Figure 1). 

These adjustments in the molecular weight were possible because all the peptides have hydrophilic character with grand average of hydropathicity (GRAVY) values ranging between −1.333 and −0.143 [21], except for L9–R21, which is hydrophobic (GRAVY of 2.031). Among the peptides identified in the hydrolysate mixture, two peptides arising from collagen type I α1 might be instable (S1221–K1236 with instability indexes of 117.86, and G695-K727 with instability indexes of 44.35), whereas all others can be characterized as stable, having the instability index values lower than 40 (Table 1). 

The antimicrobial activity of all peptides was checked against the content of the Antimicrobial Peptide Database (APD http://aps.unmc.edu/AP/), and a similarity percentage of 54.16% of the oligopeptide G458-R478 with AP02346 entry was found, indicating potential Anti Gram+ activity [22]. According to Chakchouk-Mtibaa et al. [22], the AP02346 is able to exert antilisterial activity at a minimum concentration of 400 AU/mL.

### 3.2. Characterization of the Flavonoid Extract from Onion Skins

The extract obtained from the yellow onion skins was characterized in terms of total flavonoids content (230.31 ± 3.05 mg quercetin equivalents/g dry weight (DW)), total polyphenols content (98.73 ± 2.70 mg gallic acid equivalents/g DW), and antioxidant activity (101.69 ± 0.53 mMol Trolox/g DW). Munir et al. [23] used subcritical water to extract bioactive compounds from waste onion skin and reported values ranging from 43.35 to 114.84 mg quercetin equivalents/g DW, as a function of temperature, particle sizes, and pH. The same parameters influenced the antioxidant activity with a variation between 277.89 and 698.34 mMol/g DW. Using a combination of intense pulsed light and subcritical water extraction, Kim, Ko, and Chung [24] reported a maximum concentration of quercetin of 17.32 ± 1.12 mg/g onion skin at the subcritical water extraction condition of 145 °C for 15 min with intense pulsed light at 1200 V for 60 s, whereas the quercetin concentration was 15.19 ± 1.12 mg/g onion skin using subcritical water extraction alone at 145 °C for 15 min.

### 3.3. Quenching of Peptides Solutions with Flavonoids Extract

**Figure 2:**
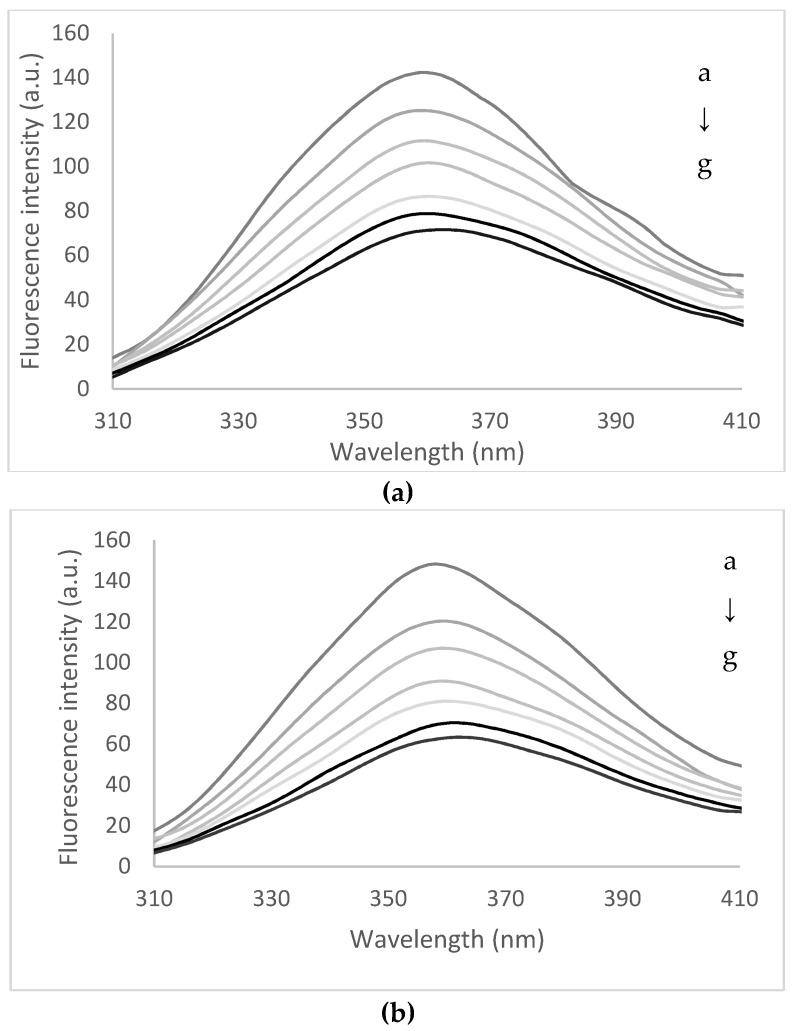
The fluorescence spectra of the interaction between fish waste low molecular peptides thermally treated at 25 °C (**a**) and 95 °C (**b**) and flavonoids from yellow onion skins. The flavonoids concentration (from a–g) varied from 0 to 1.40 × 10^−4^ M.

The fluorescence quenching process generally refers to any process that decreases the fluorescence intensity of a given compound or fluorophore through the interaction with a specific chemical or physical agent, which is known as a quencher [25]. Ciotta, Prosposito, and Pizzoferrato [26] emphasized that in the case of a particularly fluorophore embedded in a relatively complex molecule or aggregate, such as a protein, interacting with a chemical quencher, such as a certain molecule, is of great interest for both fundamental research and applications. In this study, the quenching of peptides resulted from the fish waste hydrolysis with papain were quenched with flavonoids from yellow onion skins extract in order to obtain a detailed description of the binding mechanism and the main forces involved in interactions, as a prerequisite for microencapsulation. Additionally, the investigation of molecular structure-binding affinity or biological activity relationship between active small molecules and target peptides is of great importance for understanding the bioactives–protein interaction and accelerating their applications [27]. Therefore, in the first step, the peptide solutions were heated in the temperature range of 25 to 95 °C for 15 min. At 25 °C, the peptides showed a maximum fluorescence intensity at wavelength (λ*_max_*) of 359 nm, whereas heating caused a small 2 nm red-shift, suggesting an unfolding at 85 °C. The fluorescence quenching effect exerted by flavonoids from yellow onion skins extract on fish waste peptides in buffer solution has been investigated for a broad range of fluorophore/quencher concentrations. Therefore, the fish waste peptides of low molecular weight were used for flavonoid extract quenching experiments with a concentration varying between 0 and 1.40 × 10^−4^ mol. As displayed in Figure 2, the addition of the extract progressively decreased the fluorescence intensity of the peptides at 25 and 95 °C, without appreciable changes of the spectral profile, with a typical fluorescence quenching effect. The extract did not present a distinct intrinsic fluorescence peak under the same assay conditions (data not shown). Therefore, at 25 °C, increasing the flavonoid concentration caused a significant 3 nm red-shift in λ*_max_* and a maximum quenching effect in fluorescence intensity of approximatively 60%. When the peptides were heated at 75 °C and quenched with increasing concentration of extract (1.40 × 10^−4^ M), the binding of the flavonoids was evidenced by 6 nm red-shifts in λ*_max_* at the same quenching effect. The effect was less evidenced at higher temperature, when a folding effect appeared, which was suggested by a red-shift of only 2 nm, whereas the decrease in fluorescence intensity was of approximately 62% and 64% at 85 and 95 °C, respectively. These acquired data demonstrated that the flavonoids from the extract could interact with peptides and effectively induce the fluorescence quenching effect.

The experimental Stern–Volmer plot exhibited a linear behavior; thus, a dominant static quenching with a linear plot is expected according to the classical Stern–Volmer equation. For all the tested temperatures, the plots showed *R^2^* values higher than 0.98, suggesting that the measured *K_SV_* values and *K_q_* were acceptable and reliable. The linearity of the Stern–Volmer plots suggest that there is one class of fluorescence quenching mechanism (either static or dynamic) occurring for the interaction between peptides from fish waste and bioactive compounds from yellow onion skins extract. The fluorescence Stern–Volmer, binding constants, and the number of binding sites are shown in Table 2. The *K_SV_* values ranged from 9.03 ± 0.42 × 10^4^ mol^−1^ at 25 °C and increased with increasing temperature up to 12.19 ± 0.61 × 10^4^ mol^−1^ at 95 °C. These values obtained at different temperatures suggest that the flavonoids could cause different fluorescence quenching rates on the peptides and ultimately quench its intrinsic fluorescence, which was ascribed to the difference of structural elements or substituents of flavonoid scaffolds, influencing the binding patterns in the active site of the peptides. Considering the calculated *K_q_* (Table 2) in comparison with a maximal scatter collision quenching constant (2.0 × 10^10^ L/mol·s), it can be appreciated that the static quenching mechanism mainly dominated the interaction process between these bioactives from the extract and peptides coupled with the formation of a stable flavonoid–peptides complex [28].

The binding constants of peptides with bioactives from the extract were found to decrease with increasing temperature, from 1.10 ± 0.11 × 10^6^ mol^−1^ at 25 °C to 0.92 ± 0.01 × 10^6^ mol^−1^ at 95 °C. However, the distribution of the binding values in the range of 10^6^ suggests a considerable binding potency within the active site of fish waste peptides. Additionally, as listed in Table 2, the number of binding sites (*n*) were approximately equal to 1 (varying from 0.73 ± 0.08 at 25 °C and decreased to 0.65 ± 0.05 at 95 °C), suggesting the presence of one single or one class of bind site during the interaction process of flavonoids and peptides. 

Thermodynamic parameters allowed estimating the main forces involved in interactions between ligands and peptides (Table 3). 

The negative values of Δ*H* and Δ*S* suggest that the main forces involved are hydrogen bonds and van der Waals interactions. The values of thermodynamic parameters allowed estimating that the binding process is entropy driven. The negative values of Δ*G*^0^ at 25 °C suggested that the binding of flavonoids on peptides appeared as a spontaneous process (Table 3). Comparing the values of Δ*G*^0^ is fair to affirm that the binding capacity decreased with increasing temperature, which was consistent with the determined binding constants.

### 3.4. Microencapsulation of the Flavonoidic Extract in Bioactive Fish Peptides

Microencapsulation is a widely used technology to improve the stability and bioavailability of bioactive compounds, such as flavonoids [29]. Probably the most commonly used technique for the microencapsulation of bioactives is spray drying, due to its high cost-efficiency, flexibility, and continuous production [30]. However, freeze drying was chosen in this study as being a more protective technique for sensitive and expensive bioactive compounds, since this technique uses a much lower temperature and operates in the absence of oxygen. Therefore, in order to improve the susceptibility of flavonoids from yellow onion skins extract to environmental stresses such as oxygen, light, and elevated temperature, which are generally associated with processing, a freeze-drying microencapsulation was applied, using as coating materials fish waste peptides and inulin/chitosan as adjuvants. A mixture containing 0.1 g flavonoids/g peptides was subjected to freeze-drying. A fine light orange-brown powder was obtained with an encapsulation efficiency for total flavonoids of 88.68 ± 2.37%. The phytochemicals present in the powder includes total flavonoids of 75.72 ± 2.58 quercetin equivalents/g DW and total polyphenols of 97.32 ± 2.80 gallic acid equivalents/g DW, with antioxidant activity of 1.01 ± 0.04 mMol Trolox/g DW. A lower value for the encapsulation of tea polyphenols of 70.98% in hydroxypropyl methylcellulose phthalate was reported by Wang, Li, Chen, Liu, and Chen [31], whereas Milea et al. [16] encapsulated yellow onion skins extract in a biopolymeric complex formed by maltodextrin/pectin/whey proteins hydrolysates in a ratio of 2:1:0.4, suggesting lower values of 66.46 ± 0.18%. These authors reported a phytochemicals profile of the powder consisting in flavonoids of 69.26 ± 1.03 mg quercetin equivalents/g DW, total polyphenols of 101.11 ± 0.47 mg gallic acid equivalents/g DW, and a significant antioxidant activity of 337.57 ± 0.89 mM Trolox/g DW.

### 3.5. In Vitro Cytotoxicity 

The results of the MTT test showed that the microencapsulated sample was cytocompatible in a wide range of concentrations between 0.05 and 1.5 mg/mL (Table 4). Calculated values of cell viability were between 90 and 97% for all tested concentrations, similar to untreated cells (control, 100%). The highest cell viability value (97.22%) was registered at the 0.05 mg/mL encapsulated sample, and a concentration-dependent decrease of cell viability was observed. 

**Figure 3:**
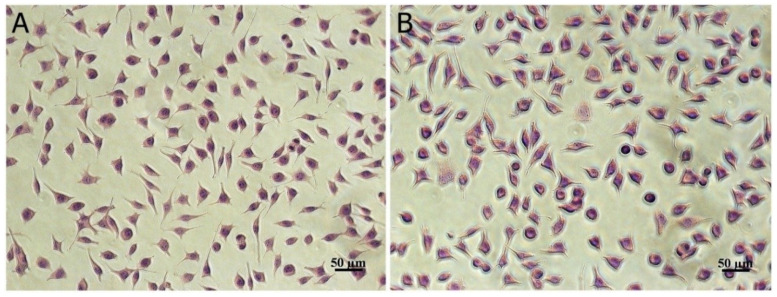
Light micrograph of L929 fibroblasts treated with 0.05 mg/mL microencapsulated sample (**B**) indicating similar cell morphology to untreated control cells (**A**), after 24 h of cultivation in standard conditions. Giemsa staining. Scale bar = 50 µm.

According to ISO 10993-5 [18] for medical device cytotoxicity testing, samples that give a cell viability higher than 80%, as in case of our study, are not cytotoxic. Light micrographs of fibroblast cells cultivated in the presence of microencapsulated sample confirmed MTT results, indicating no signs of cytotoxicity. The treated cells maintained a normal morphology, specific to fibroblasts, with a fusiform shape and clear cytoplasm, similar to untreated control cells (Figure 3).

Previous studies reported no cytotoxicity of vegetable, fruit, or grain extracts encapsulated with food proteins/peptides, although a dose–response pattern was registered in different cell lines. Thus, citron extract encapsulated in fish peptides was not cytotoxic for NIH-3T3 fibroblast or HepG2 epithelial cells, even at high concentrations, up to 5 mg/mL, but in H9C2 myoblastic cells, the cytocompatibility was observed only up to 1 mg/mL [32]. Anthocyanins extract from black rice encapsulated in whey protein hydrolysate were cytocompatible in L929 fibroblast cells up to 1 mg/mL [33]. A formula based on casein was optimized for the flavonoid extract of onion peels to improve bioavailability in food applications, but no cytotoxicity studies were found [34].

## 4. Conclusions

In this study, a bidirectional approach was taken for bioprospecting targeted applications of two different food by-products, namely fish waste and yellow onion skins, for efficient formulations in order to increase the health-promoting properties. Therefore, bone tissue from phytophagous carp (*Hypophthalmichthys molitrix*) was used to obtain low molecular bioactive peptides through papain-assisted hydrolysis, which were characterized through MALDI-ToF/ToF mass spectrometry and bioinformatics tools. More than 36 peptides with different low molecular weights were generated from papain-assisted hydrolysis of collagen type I α1 and type I α2. MALDI-ToF/ToF mass spectrometry allowed an in-depth characterization of the resulting peptides, whereas the comparison with different databases showed a similarity percentage of 55% of the oligopeptide G458-R478 with AP02346 entry, indicating potential Anti Gram+ activity.

A static quenching mechanism mainly dominated the interaction process between flavonoids and peptides, indicating a stable bioactive complex, based on hydrogen bonds and van der Waals interactions. The quenching experiments allowed estimating the presence of one single or one class of bind site during the interaction process of flavonoids and peptides. By coacervation and freeze-drying, a fine orange-brown powder was obtained, showing a significant bioactives content and antioxidant activity, indicating no signs of cytotoxicity. Given the obtained results, new perspectives are provided to advance the knowledge in the agri-food pharma-health industry for the development of novel formulation, based on bioactive compounds from natural sources, which is associated with the reliable perspectives for the reuse of underutilized bioactives-rich by-products.

## Figures and Tables

**Table 1:** Physicochemical properties of the peptides obtained through papain-assisted hydrolysis of collagen type I α1 and collagen type I α2 from *Hypophthalmichthys molitrix.*

Peptide Sequence	pI	MW	GRAVY	Instability Index
***Peptides originating from collagen type I α1***				
*812-GEAGDNGAK-820*	4.37	817.81	−1.333	−25.62
*337-GEVGPQGAR-345*	6.00	869.93	−0.922	3.04
*251-GHRGFSGLDGAK-262*	8.75	1201.31	−0.758	−4.98
*9-LALLLSATVLLAR-21*	9.75	1353.71	2.031	9.23
*1221-SLSQQIESIMSPDGTK-1236*	4.37	1720.91	−0.569	117.86
*458-GEPGAAGGRGPPGERGAPGAR-478*	9.51	1874.01	−1.09	15.52
*434-GEAGAQGVQGPPGPPGEEGKRGAR-457*	6.23	2259.42	−1.267	32.37
*308-GNDGAAGAAGPPGPTGPAGPPGFPGGPGAK-337*	5.84	2454.64	−0.507	25.31
*695-GDSGAPGAPGAQGPPGLQGMPGERGAAGLPGLK-727*	6.07	2926.26	−0.452	44.35
*1384-AEGNSRFTYSVTEDGCTSHTGAWGKTVIDYK-1414*	5.49	3381.63	−0.716	19.36
***Peptides originating from collagen type I α2***				
*1109-ADQASLRAK-1117*	8.79	959.07	−0.778	20.86
*1118-DYEVDATVK-1126*	4.03	1039.11	−0.689	−19.39
*1274-KAVLLQGSNDVELR-1287*	6.07	1541.77	−0.143	46.33
*423-GPPGDAGRAGEPGLVGAR-440*	6.07	1633.78	−0.544	17.22

pI—isoelectric point; MW—molecular weight; GRAVY—grand average of hydropathicity.

**Table 2:** The binding parameters of the fish waste low molecular weight peptides heat treated at different temperatures by flavonoids extracts from yellow onions skins.

T (°C)	*K_SV_* (10^4^ mol^−1^)	*K_q_* (10^14^ L/mol^−1^ s^−1^)	*K_b_* (10^6^ mol^−1^)	*n*
25	9.03 ± 0.42 ^b,1^	9.03 ± 0.42 ^b^	1.10 ± 0.11 ^a^	0.73 ± 0.08 ^a^
75	9.52 ± 0.34 ^b^	9.52 ± 0.34 ^b^	1.01 ± 0.03 ^a,b^	0.72 ± 0.01 ^a^
85	11.80 ± 0.43 ^a^	11.80 ± 0.43 ^a^	0.97 ± 0.02 ^a,b^	0.69 ± 0.02 ^a^
95	12.19 ± 0.61 ^a^	12.19 ± 0.61 ^a^	0.92 ± 0.01 ^b^	0.65 ± 0.05 ^a^

^1^ Mean values within a column that do not share a superscript letter (^a^ or ^b^) are significantly different at *p* < 0.05.

**Table 3:** The thermodynamic parameters for the association fish waste low molecular weight peptides heat and flavonoids extracts from yellow onions skins.

T(K)	Δ*H*^o^ (J·mol^−1^)	Δ*S*^o^ (J·mol^−1^·K^−1^)	Δ*G*^o^ (J·mol^−1^)	*R* ^a^
298	−251.02 ± 11.45 ^a^	−0.73 ± 0.08	−32.31 ± 1.25	0.92
348			4.37 ± 1.12	
358			11.71 ± 0.97	
368			19.05 ± 1.90	

^a^—standard errors.

**Table 4:** Values and statistical analysis of L929 cell viability after cultivation in the presence of different concentrations of microencapsulated samples, for 24 h, determined by 3-(4,5-dimethylthiazol-2-yl)-2,5-diphenyltetrazolium bromide (MTT) assay.

Sample	Control	Microencapsulated Sample					H_2_O_2_
Concentration (µg/mL)	-	50	100	500	1000	1500	1.7
Cell viability (%)	100	97.22	95.43 *	93.10 **	90.77 **	89.96 **	7.97 **
Degree of cytotoxicity	-	NC	NC	NC	NC	NC	C
SD	1.17	1.48	1.94	1.95	1.62	2.19	0.41
*p*	-	0.063	0.025	0.006	0.001	0.002	0

NC—not cytotoxic, C—cytotoxic, * *p* < 0.05, ** *p* < 0.01 compared to control.
